# SNHG9, a Papillary Thyroid Cancer Cell Exosome-Enriched lncRNA, Inhibits Cell Autophagy and Promotes Cell Apoptosis of Normal Thyroid Epithelial Cell Nthy-ori-3 Through YBOX3/P21 Pathway

**DOI:** 10.3389/fonc.2021.647034

**Published:** 2021-05-04

**Authors:** Duo Wen, Wan-lin Liu, Zhong-wu Lu, Yi-ming Cao, Qing-hai Ji, Wen-jun Wei

**Affiliations:** ^1^Department of Head and Neck Surgery, Fudan University Shanghai Cancer Center, Shanghai, China; ^2^Department of Oncology, Shanghai Medical College, Fudan University, Shanghai, China

**Keywords:** SNHG9, papillary thyroid cancer, exosome, autophagy, apoptosis, lncRNA

## Abstract

Thyroid cancer is the most common type of endocrine malignancy. Although the general prognosis is good, the treatment of advanced disease is still challenging. Exosomes are vesicle units containing specific components that transmit information between cells. In order to explore its role in papillary thyroid cancer (PTC), our study screened exosome enriched lncRNA SNHG9 by lncRNA chip and explored its biological function. We used lncRNA chips combined with bioinformatics analysis to screen lncRNA SNHG9 enriched in exosomes. GO analysis suggested its relationship with autophagy and apoptosis. Quantitative PCR showed SNHG9 was highly expressed in PTC cells and exosomes and its correlation with PTC tumor size was analyzed by clinical characteristics. SNHG9 could inhibit the protective cell autophagy induced by starvation of human normal thyroid epithelial cell line Nthy-ori-3 and promote its apoptosis through PTC cell exosomes. RNA-pull down combined with protein spectrum showed that SNHG9 could interact with YBOX3. Western blot and RNA immunoprecipitation further confirmed their interaction. Western blot showed that SNHG9 could induce degradation of YBOX3, thus interfering with the stability of P21 mRNA and inducing cell apoptosis. In conclusion, our study identified SNHG9 as a PTC cell exosome-enriched lncRNA. SNHG9 could inhibit cell autophagy and promote apoptosis of Nthy-ori-3 cell through YBOX3/P21 pathway.

## Introduction

Thyroid cancer is the most common type of endocrine malignancy and according to recent data, ranks as the fifth most common cancer in women in the USA (1). Papillary thyroid carcinoma (PTC) comprises 80% of all thyroid cancers (2). Although the prognosis of PTC is usually excellent, with 5- and 10-year survival rates exceeding 95% and 90%, respectively, approximately 20% of PTC patients develop recurrent disease (3). These diseases include local cervical recurrence and distant metastasis, which may be incurable and fatal.

Exosomes refer to a class of vesicles approximately 30-150 nm in size (due to the limitation of purification methods, some collectively refer to units smaller than 200 nm as small extracellular vesicles) that originate from intracellular multivesicles (multivesicular bodies, MVBs) and are released after fusion with cell membranes (4, 5). These subcellular vesicle-like structures that are actively secreted by cells are surrounded by a lipid bilayer membrane. In recent years, a series of component analyses have found that exosomes carry a large cargo of proteins, lipids, nucleic acids as well as other components from mother cells, and a considerable proportion of them exhibit tissue and cell specificity (6). As increasingly more functions of exosomes are revealed, new interpretations of long-discussed issues in the field of oncology are being presented from a new perspective. For example, Hoshino et al. found that some tumor cell-derived exosomes can selectively increase uptake by specific distant organs, causing the tissue microenvironment to be more suitable for the survival and development of tumor cells and promoting migration of tumor cells to the liver, lung, brain and other organs (7). In renal cell carcinoma, sunitinib-resistant tumor cells can deliver specific long noncoding RNAs (lncRNAs) *via* exosomes, conferring resistance to sunitinib on surrounding nonresistant cells (8). This finding successfully confirmed the important role that exosomes play in the spread of tumor drug resistance through the cellular microenvironment. Concomitantly, new targets have emerged in research related to overcoming tumor drug resistance.

LncRNAs are noncoding RNAs consisting of >200 nucleotides (9). Increasing studies have reported that lncRNAs play vital roles in cancer cell proliferation, metastasis and chemoresistance (10). Moreover, lncRNAs tend to exhibit higher tissue-specific expression than protein coding genes. Although the expression specificity of lncRNAs provides opportunities for exploring new biomarkers and drug targets, the molecular mechanism of lncRNAs remains poorly understood. Small nucleolar RNA host gene 9 (SNHG9) is located on chromosome 16p13.3. It has been shown to be involved in several types of cancer, such as glioblastoma (11), pancreatic cancer (12) and non-small cell lung cancer (13). However, the function of SNHG9 in papillary thyroid cancer is still unknown.

Through extensive research, exosomes have been confirmed to be closely related to the occurrence and development of a variety of malignant tumors. Nonetheless, there are few relevant studies and evidence in the field of PTC research. Indeed, the relationship between exosomes and PTC is currently unknown.

In this study, we conducted a systemic analysis of the PTC exosome-enriched lncRNA SNHG9. Our study revealed SNHG9 to be a PTC exosome-enriched lncRNA that is able to inhibit autophagy and promote apoptosis in the thyroid normal epithelial cell line Nthy-ori-3 by interfering with YBOX3 and inhibiting the P21 protein.

## Materials and Methods

### Tissue Specimens

Papillary thyroid cancer samples and corresponding normal thyroid tissues at least 1cm away from the tumor were obtained from patients who underwent thyroid cancer surgery at Fudan University Shanghai Cancer Center (FUSCC). Tissue specimens were frozen in liquid nitrogen immediately after surgical resection and stored at -80°C until use. Histological classification was obtained from paraffin-embedded sections. This study was performed in accordance with the 1964 Helsinki Declaration and its later amendments or comparable ethical standards. It was approved by the Human Ethics Committee/Institutional Review Board of Fudan University Shanghai Cancer Center.

### Cell Lines and Cell Culture

Two papillary thyroid cancer cell lines, TPC-1, K-1 and one normal human thyroid epithelial cell line Nthy-ori-3 were used for cell experiments. Nthy-ori 3 cell line was purchased from Sigma. TPC-1 and K-1 cell lines were purchased from the Cell Bank of University of Colorado. All cell lines were cultured in RPMI-1640 medium (GIBCO) supplemented with 10% heat-inactivated fetal bovine serum (GIBCO) at 37°C in a 5% CO_2_ chamber.

### Total RNA Extraction, Reverse Transcription and Quantitative Real-Time PCR Analysis

Total RNA was extracted from tissues and cultured cells using TRIzol Reagent (Invitrogen) according to the manufacturer’s instructions. RNA purity and concentration were determined by the NanoDrop2000 spectrophotometer. A total of 1 μg of RNA was reverse-transcribed using a PrimeScript RT reagent kit (Takara, Dalian, China). For quantitative real-time PCR (qPCR), cDNA was amplified using SYBR Green Premix Ex Taq (Takara, Dalian, China) following the manufacturer’s instructions. Gene expression was normalized against beta actin mRNA expression in three independent experiments. The relative mRNA expression level were determined by the comparative Ct (2^-ΔCt^)method.

### Western Blotting

Cell lysates were obtained from 1×10^6^ cultured cells with RIPA protein extraction reagent. The protein concentration was determined by bicinchoninic acid assay (BCA). Equal amounts (30μg) of total protein lysate were separated by 10% sodium dodecyl sulfate-polyacrylamide gel electrophoresis (SDS-PAGE) and then transferred onto PVDF membrane. The membranes were then blocked in 5% non-fat milk at room temperature for 1 hour. Following this treatment, the membranes were probed with primary antibodies against human P62 (1:1000 dilution, Cell Signaling Technology, USA), P21 (1:1000, Proteintech), YBOX3 (1:1000, Cell Signaling Technology, USA), Histone 3 (1:1000, Proteintech), GAPDH (1:1000, Abcam, USA), CD63 (1:1000, Proteintech), CD9 (1:1000, Proteintech), Caspase 9 (1:1000, Proteintech), Cleave caspase 9 (1:1000, Proteintech), Caspase 3 (1:1000, Proteintech), Cleave caspase 3 (1:1000, Proteintech), Caspase 7 (1:1000, Proteintech), Cleave caspase 7 (1:1000, Proteintech), Beclin1 (1:1000, Proteintech), Atg5 (1:1000, Proteintech), LCII (1:1000, Proteintech), LCIII (1:1000, Proteintech) at 4°C overnight. Following incubation in a solution of goat anti-rabbit (1:1000, Santa Cruz, USA) or anti-mouse IgG (1:5000 for both; Santa Cruz, USA) at room temperature for 1hour, the membranes were washed with TBST and then detected with enhanced chemiluminescence reagents (Thermo Fisher Scientific, Inc.). The bands were visualized using 1-step TM NBT/BCIP reagents (Thermo Fisher Scientific, Rockford, IL, USA) and detected by the Alpha Imager (Alpha Innotech, San Leandro, CA, USA).

### Exosome Isolation and Exosome Protein Extraction

The cell medium was centrifuged at 500 *g* for five minutes and at 2000 *g* for thirty minutes at 4 °C to remove cellular debris and large apoptotic bodies. After centrifugation, media was added to an equal volume of a 2× polyethylene glycol (PEG, MW 6000, Sigma, 81,260) solution (final concentration, 8%). The samples were mixed thoroughly by inversion and incubated at 4°C overnight. Before the tubes were tapped occasionally and drained for five minutes to remove excess PEG, the samples were further centrifuged at maximum speed (15,000 rpm) for 1 h at 4°C. The resulting pellets were further purified using 5% PEG and then stored in 50–100 μl of particle-free PBS (pH 7.4) at − 80°C. The average yield was approximately 300 μg of exosomal protein from 5 ml of supernatant. Exosome was re-suspended in 150ul RIPA buffer (Beyotime, P0013B) and incubated on ice for 1h. The lysate was centrifuged for 20min at 16000g at 4°C, and the supernatant was further concentrated by ultracentrifuge filter (Millipore, 3kDa cutoff). Protein concentration was measured by BCA assay (Beyotime, P0012).

### RNA-Pull Down Assay

The resultant plasmid DNA was linearized with restriction enzyme NotI. Biotin-labeled SNHG9 lncRNAs were *in vitro* transcribed with the Biotin RNA Labeling Mix (Roche Diagnostics, Indianapolis, IN, USA) and T7 RNA polymerase (Roche, Basel, Switzerland), treated with RNase-free DNase I (Roche) and purified with the RNeasy Mini Kit (Qiagen, Inc., Valencia, CA, USA). Nthy-ori-3 cell extract (2 μg) was mixed with biotinylated RNA (100 pmol). One hour after incubation at 4°C, washed streptavidin‐coupled agarose beads (Invitrogen) were added to each binding reaction and further incubated at room temperature for 1 h to isolate the RNA–protein complex. Beads were washed briefly three times and boiled in SDS buffer, and the retrieved protein was detected by standard western blot technique.

### RNA Immunoprecipitation (RIP) Assay

RIP assays were performed using an EZ-Magna RIP™ RNA-Binding Protein Immunoprecipitation Kit (Millipore, Billerica, MA, USA) according to the manufacturer’s instructions. Cells at approximately 90% confluence was lysed using complete RIP lysis buffer containing RNase Inhibitor (Millipore) and protease inhibitor and then 100 μl of whole cell extract was incubated with RIP buffer containing magnetic beads conjugated to specific antibodies. The negative control was normal mouse anti-IgG antibody (Cell Signaling Technology, USA).

### McHerry-gfp-lc3 Autophagy Flow Detection

Cells cultured in 24-well plates (1×105 cells/well) were transduced with mCherry-GFP-LC3 adenovirus at 40 MOI (multiplicity of infection) for 24 h at 37°C in a humidified atmosphere containing 5% CO2/95% air. Following transduction, the cells were incubated with fresh culture medium for 24 h at 37°C. The numbers of GFP and mCherry dots per cell were counted in three randomly selected fields under a fluorescence microscope.

### TUNEL Staining Assay

The treated cells were rinsed with PBS twice and fixed in 4% paraformaldehyde. The endogenous peroxidase activity was blocked by methanol followed by cell permeabilization with a cocktail of 1 g/L TritonX-100 in 0.1% sodium citrate. After washing with PBS, all sections were incubated with TUNEL mixture for 60 min, followed by an incubation with DAPI for 10 min. Finally, the tissue sections were observed with a confocal microscopy.

### Cell Apoptosis Analysis by Flow Cytometry

Treated cells were trypsinized. The resuspended cells were washed with PBS and stained with annexin V‐FITC and propidium iodide (PI) according to the instructions of AnnexinV‐FITC apoptosis detection kit (BD Biosciences, USA). Cells were then analyzed by flow cytometry (CytoFLEX; Beckman Coulter, Brea, CA, USA).

### Statistics Analysis

Statistical analyses were performed with SPSS version 19.0 for Windows. For comparison among the groups, a Student’s *t* test, Chi-Square test or a Fisher’s Exact test was performed, and *P*<0.05 was defined as statistically significant. The data and error bars report the means ± SEM. Each experiment was repeated at least three times.

## Results

### SNHG9 Is Enriched in and Protected by PTC Cell Exosomes

To evaluate PTC exosomal-specific lncRNAs, we first isolated exosomes from two PTC cell lines, TPC-1 and K-1, and one normal thyroid epithelial cell line, Nthy-ori-3, and detected expression of the exosome-specific protein markers CD9 and CD63 (14) by Western blotting (**Supplementary Figure 1A**). In addition, exosome morphology was observed by transmission electron microscopy. The shape of various sizes of exosomes was round or elliptical, displaying their typical “saucer-like” structure (**Supplementary Figures 1B, D**). Combining the characteristics of protein detection and electron microscopy morphological identification, we confirmed that the exosome extraction method adopted in this study could successfully extract exosomes from PTC cells.

**Figure 1:**
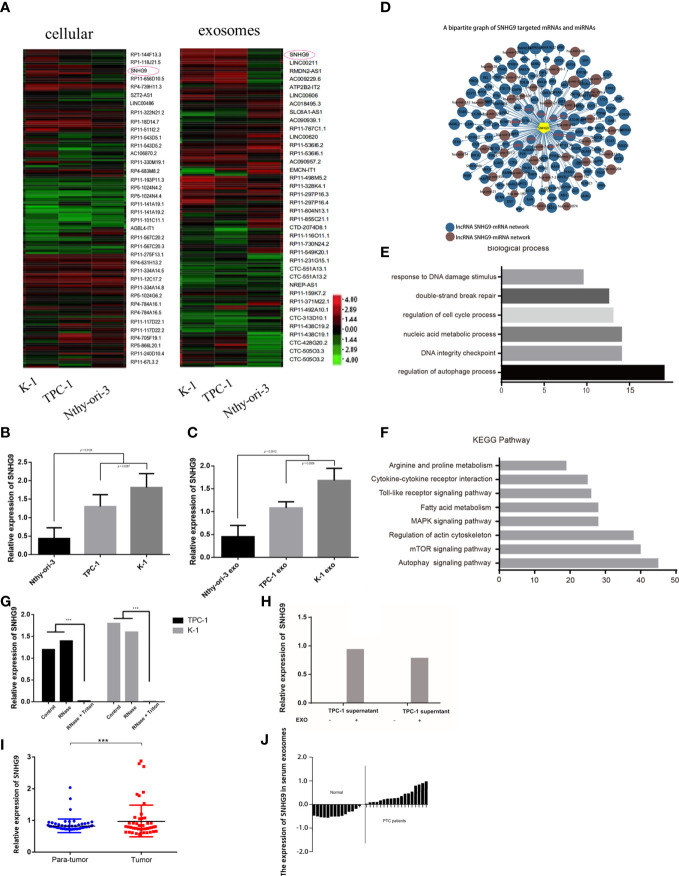
Identification and expression of PTC cell exosome-enriched lncRNA SNHG9. **(A)** High-throughput screening identification of PTC associated exosome lncRNAs. SNHG9 is PTC cell exosome-enriched lncRNA in TPC-1 and K-1 cells compared with Nthy-ori-3 cell. **(B, C)** Validation of SNHG9 overexpression in both TPC-1 and K-1 cells and their respective exosomes compared with Nthy-ori-3 cell and its exosome by qPCR. **(D)** Coregulation network of SNHG9 with mRNA/miRNA. SNHG9 had an interaction with autophagy related molecules. **(E)** Gene ontology enrichment analysis showed the highest regulation scores in autophagy and apoptosis. **(F)** KEGG-pathway-weighted analysis showed SNHG9 mainly targeted apoptosis and autophagy pathways. **(G)** SNHG9 in the PTC cell supernatant mainly derived from cell exosomes. QPCR showed significantly lower SNHG9 expression level in supernatant treated with Rnase and Triton compared with supernatant treated with only Rnase and control group. **(H)** QPCR confirmed no SNHG9 expression in cell supernatants after exosome extraction. **(I)** SNHG9 expression level between tumor and normal tissues in 70 PTC patients from FUSCC. The results were normalized to β-actin mRNA level. **(J)** Waterfall plot showed the distribution of SNHG9 expression level in each PTC patients from FUSCC. ***P < 0.001, data were pooled from three independent experiments. FUSCC, Fudan University Shanghai Cancer Center; PTC, papillary thyroid cancer.

Next, we used the Arraystar Human LncRNA Array v2.0 gene chip to compare expression profile data of lncRNAs in Nthy-ori-3, TPC-1 and K-1 cells and their respective exosomes. A total of 2804 known lncRNAs were identified in PTC cell exosomes. Among them, the expression levels of 172 and 253 known lncRNAs in exosomes derived from TPC-1 and K-1 cell lines were significantly upregulated, respectively, and 369 and 418 were significantly downregulated, respectively. Compared with Nthy-ori-3, lncRNA SNHG9 showed specifically high expression in PTC cell lines and their exosomes (**Figure 1A**). The expression of SNHG9 levels were analyzed in three cell lines and their respective exosomes by qPCR. **Figures 1B, C** show that in both the cells and secreted exosomes, expression of SNHG9 was significantly higher in TPC-1 and K-1 cells than in the normal thyroid epithelial cell line Nthy-ori-3.

The prediction results based on sequence specificity showed rich interactions between SNHG9 and other genetic molecules. These interactions can be summarized into two main regulatory networks: the SNHG9-mRNA network and the SNHG9-miRNA network. Regarding the former, we found that autophagy-related molecules, including DRAM1, BECN1 and the ATG family, were able to interact with SNHG9 (**Figure 1D**). Based on GO analysis, SNHG9 is involved in DNA damage repair, cell cycle regulation and other processes, among which autophagy and apoptosis regulation had the highest scores (**Figure 1E**). Furthermore, we used KEGG signal pathway weighted analysis and found that SNHG9 mainly targets apoptosis and autophagy pathways (**Figure 1F**), further suggesting involvement of SNHG9 in the regulation of PTC cell autophagy and apoptosis.

To clarify whether the lncRNA SNHG9 present in the cell culture supernatant was derived from exosomes secreted by PTC cells, we treated the supernatant with either RNase or RNase plus Triton 100, which could disrupt the exosomal membrane. According to qPCR, the abundance of SNHG9 in exosomal samples treated with RNase alone was unchanged compared with the control group, though its abundance was significantly lower in the RNase plus Triton 100 group (**Figure 1G**). This illustrates the protective effect of exosomes on SNHG9. As depicted in **Figure 1H**, we could not detect SNHG9 in TPC-1 and K-1 cell supernatants after exosome extraction, which further confirmed the enrichment of SNHG9 in exosomes.

### SNHG9 Is Overexpressed in Human PTC Tissues

We also used qPCR to explore SNHG9 expression levels in 70 pairs of PTC and adjacent thyroid specimens from the FUSCC cohort, and the results showed SNHG9 to be upregulated in PTC samples (**Figure 1I**). The correlation of SNHG9 with the clinicopathological characteristics of PTC patients is presented in **Table 1**. High SNHG9 expression was significantly associated with a large tumor size (P=0.034) but did not correlate with tumor invasion, lymph node metastasis or other variables. Therefore, SNHG9 is overexpressed in human PTC tissues and is related to tumor malignancy, suggesting that SNHG9 plays an oncogenic role in PTC.

**Table 1:** The correlation of SNHG9 with the clinicopathological characteristics of PTC patients in the FUSCC.

Clinical parameters	SNHG9 expression		P-value
	High (%)	Low (%)	
Age(years)			0.325
<55	13 (55.2)	19 (41.3)	
≥55	11 (44.8)	27 (58.7)	
Gender			0.534
Male	6 (20.7)	8 (17.5)	
Female	18 (79.3)	38 (82.5)	
Tumor size (cm)			0.034*
≤1	20 (37.9)	26 (60.3)	
>1	4 (62.1)	20 (39.7)	
Location of the primary tumors Solitary lesion			0.579
Upper third	3 (12.5)	10 (21.7)	
Middle third	13 (54.2)	20 (43.5)	
Lower third	4 (16.7)	11 (23.9)	
Isthmus	4 (16.7)	5 (10.9)	
Extrathyroidal extension			0.406
Positive	2 (8.3)	6 (13.0)	
Negative	22 (91.7)	40 (87.0)	
Lymph node metastasis			0.992
Positive	11 (45.8)	22 (47.8)	
Negative	13 (54.2)	24 (52.2)	

*P < 0.05, Chi-squared test P-value.

### SNHG9 Inhibits Nthy-ori-3 Cell Autophagy Through PTC Exosomes

**Figure 2:**
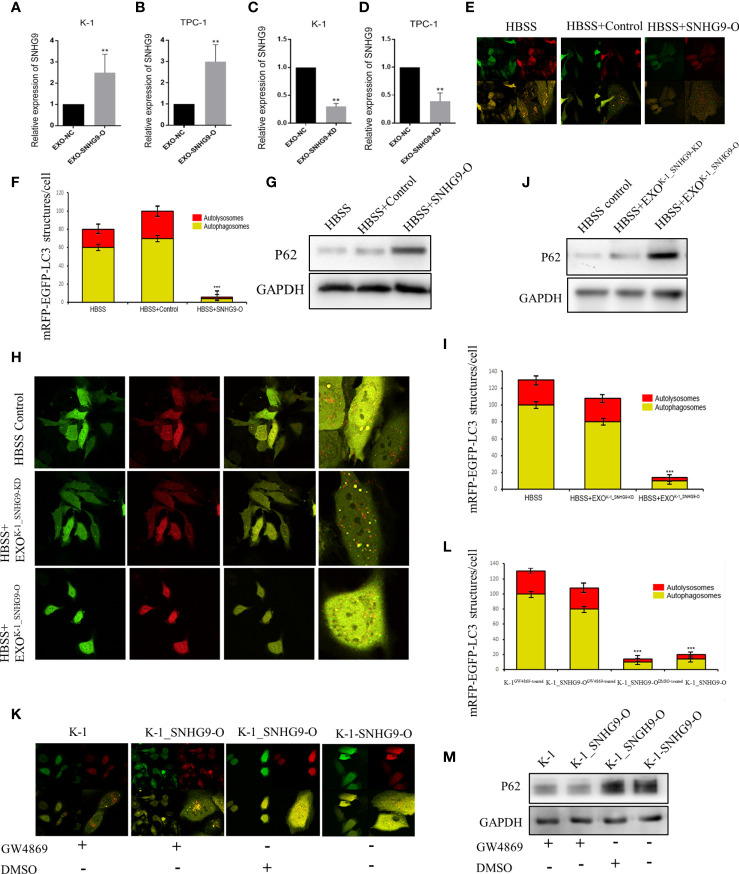
SNHG9 inhibits Nthy-ori-3 cell autophagy through PTC exosomes. **(A, B)** SNHG9 was significantly upregulated in K-1 and TPC-1 cell exosomes after overexpressing SNHG9 in PTC cells. **(C, D)** SNHG9 was significantly downregulated in K-1 and TPC-1 cell exosomes after knocking down SNHG9 in PTC cells. **(E, F)** Overexpressing SNHG9 in HBSS treated Nthy-ori-3 cells could inhibit cell autophagy detected by mRFP/mCherry-GFP-LC3B autophagy flow. Yellow spots indicate autophagosomes and red spots indicate autophagic lysosomes. **(G)** Autophagy-related protein P62 was significantly increased after overexpressing SNHG9 in HBSS treated Nthy-ori-3 cells by Western blot. **(H and I)** K-1^SNHG9-O/^K-1^SNHG9-KD^ exosomes could inhibit/promote Nthy-ori-3 cells autophagy detected by mRFP/mCherry-GFP-LC3B autophagy flow. Yellow spots indicate autophagosomes and red spots indicate autophagic lysosomes. **(J)** P62 was significantly increased in K-1^SNHG9-O^ exosome treated Nthy-ori-3 and was downregulated in K-1^SNHG9-KD^ exosome treated Nthy-ori-3 cells by Western blotting. **(K, L)** The impact of K-1^SNHG9-O/^K-1^SNHG9-KD^ exosomes on Nthy-ori-3 cell autophagy was decreased after K-1 cell exosomes secretion was inhibited by GW4869. **(M)** P62 protein change after K-1 cell exosomes secretion was inhibited by GW4869. ***P < 0.001, **P < 0.01, data were pooled from three independent experiments. PTC, papillary thyroid cancer; KD, knock down; O, overexpression. HBSS, Hank’s balanced salt solution.

Next, we investigated the biological roles of SNHG9 in PTC oncogenesis by overexpressing and knocking down SNHG9 expression *via* lentivirus transfection. QPCR assays confirmed that SNHG9 was successfully overexpressed (SNHG9-O) or silenced (SNHG9-KD) in TPC-1, K-1 and Nthy-ori-3 cells compared with negative controls (**Supplementary Figure 2**), and the data in **Figure 2** indicate that SNHG9 expression was also significantly upregulated or downregulated in exosomes (EXO-SNHG9-O/EXO-SNHG9-KD) of TPC-1 and K-1 cells.

After exogenously overexpressing SNHG9 in Nthy-ori-3 cells, we used the mRFP/mCherry-GFP-LC3B tandem fluorescent protein to detect the level of autophagy induced by Hank’s balanced salt solution (HBSS) starvation. After 2 hours of HBSS starvation induction, obvious yellow (autophagosomes) and red (autophagic lysosomes) foci in the control cells were detected (**Figures 2E, F**), indicating normal autophagy. In contrast, the red and yellow fluorescence was diffusely distributed in the cytoplasm of the cells overexpressing SNHG9, with no yellow or red foci observed. Moreover, P62 protein expression was significantly increased compared to the control group (**Figure 2G**), indicating that overexpression of SNHG9 inhibits Nthy-ori-3 cell autophagy.

We extracted K-1^SNHG9-O^, K-1^SNHG9-KD^, TPC-1^SNHG9-O^, and TPC-1^SNHG9-KD^ exosomes and their respective control groups and co-cultured them with Nthy-ori-3 cells. At 2 hours after HBSS treatment, yellow and red spots were found in the Nthy-ori-3 cells added to the control group (HBSS treated) and K-1^SNHG9-KD^ exosomes (HBSS+EXO^K-1_SNHG9-KD^), indicating that normal autophagic flow. Conversely, significantly decreased autophagosome and autophagic lysosome numbers among the Nthy-oir-3 cells from the K-1^SNHG9-O^ group (HBSS+EXO^K-1_SNHG9-O^) were observed, indicating that autophagic flow was blocked (**Figures 2H, I**). In addition, the significantly higher level of P62 protein in the HBSS+EXO^K-1_SNHG9-O^ group suggested that Nthy-ori-3 cell autophagy was inhibited (**Figure 2J**). We also observed the same phenomenon in the TPC-1 cell group (**Supplementary Figure 3**). Therefore, SNHG9 inhibits Nthy-ori-3 cell autophagy through PTC exosomes.

Next, we used a Transwell coculture system of living cells to confirm that the SNHG9-mediated regulation of Nthy-ori-3 cell autophagy is exosome dependent. Nthy-ori-3 cells were spread onto a 24-well glass bottom dish, and K-1, K-1_SNHG9-O^GW4869^-treated, K-1_SNHG9-O^DMSO^-^treated^ and K-1-SNHG9-O cells were spread onto the upper chamber. In the GW4869 (an exosome inhibitor)-treated group, the number of autophagosomes formed increased significantly (**Figures 2K, L**), and the P62 protein level decreased (**Figure 2M**), indicating normal Nthy-ori-3 cell autophagy; the same was observed in the TPC-1 cell group. These experimental results indicate that the regulation of Nthy-ori-3 cell autophagy by SNHG9 is dependent on exosomes.

### SNHG9 Promotes Nthy-ori-3 Cell Apoptosis Through PTC Exosomes

**Figure 3:**
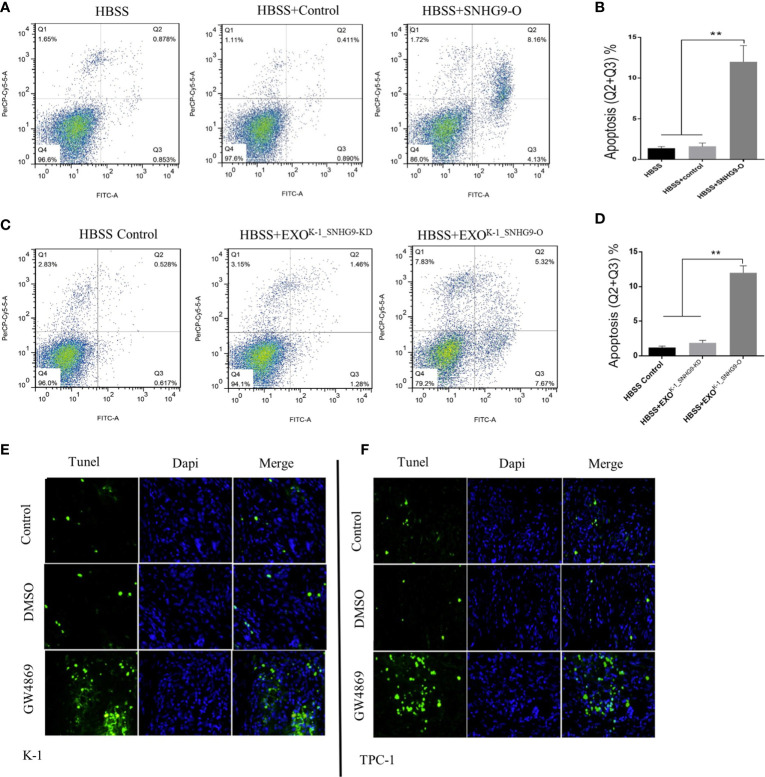
SNHG9 promotes Nthy-ori-3 cell apoptosis through PTC exosomes. **(A, B)** Overexpressing SNHG9 in HBSS treated Nthy-ori-3 cells could promote cell apoptosis detected by flow cytometry. **(C, D)** K-1^SNHG9-O/^K-1^SNHG9-KD^ exosomes could promote/inhibit Nthy-ori-3 cells apoptosis detected by flow cytometry. **(E, F)** The impact of K-1^SNHG9-O/^TPC-1^SNHG9-KD^ exosomes on Nthy-ori-3 cell apoptosis was decreased after PTC cell exosomes secretion was inhibited by GW4869 by Tunel staining assay. **P < 0.01, data were pooled from three independent experiments. PTC, papillary thyroid cancer. KD, knock down; O, overexpression; HBSS, Hank’s balanced salt solution.

Flow cytometry was then applied to ascertain the effect of SNHG9 on Nthy-ori-3 cell apoptosis. **Figures 3A, B** shows that compared with the control group, the number of Nthy-ori-3 apoptotic cells in early and late stages was significantly increased after overexpressing SNHG9. Therefore, SNHG9 promotes Nthy-ori-3 cell apoptosis.

Next, using the exosomes and Nthy-ori-3 coculture system mentioned above, flow cytometry results indicated significant increases in the number of Nthy-ori-3 apoptotic cells in the SNHG9-enriched exosome group (HBSS+EXO^K-1_SNHG9-O^) compared to the HBSS+EXO^K-1_SNHG9-O^ and HBSS-treated groups (**Figures 3C, D**). We obtained the same results for the TPC-1 exosome-treated groups (**Supplementary Figure 4**).

We also used TUNEL staining to detect the level of Nthy-ori-3 cell apoptosis in the Transwell coculture system of living cells. As illustrated in **Figures 3E, F**, compared with the control group, the number of apoptotic cells in early and late stages was obviously increased after exosome inhibition by GW4869. All these results suggest that SNHG9 promotes Nthy-ori-3 cell apoptosis in a PTC cell exosome-dependent manner.

### SNHG9 Interacts With YBOX3

**Figure 4:**
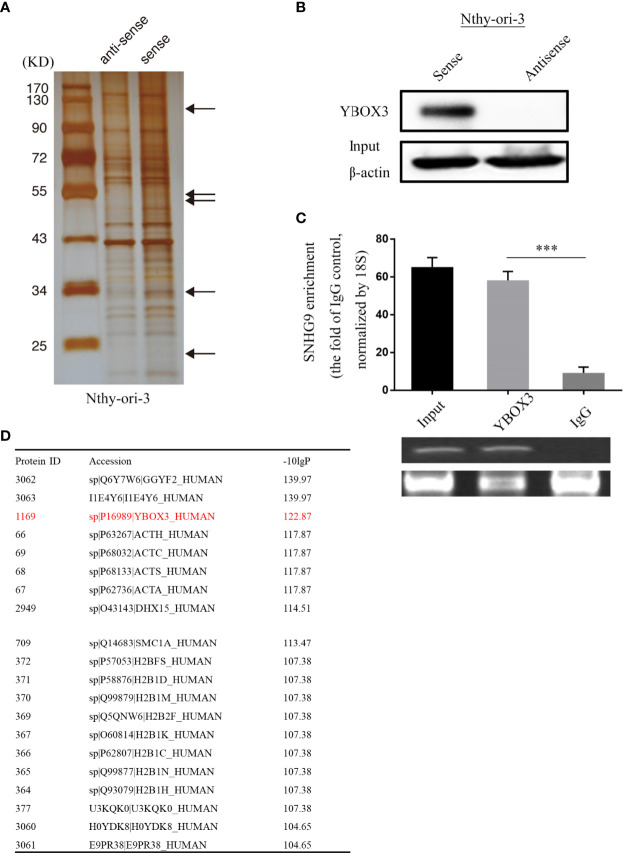
SNHG9 interacts with YBOX3 in Nthyi-ori-3 cell. **(A)** An RNA-pull down assay was performed and SNHG9 related proteins were determined with SDS-PAGE and silver staining. **(B)** YBOX3 was pulled down by a SNHG9 sense RNA probe but not by an antisense RNA probe in Nthyi-ori-3 cell. **(C)** RIP assays with qPCR (top) or RT-PCR (low) showed that SNHG9 was pulled down by an anti-YBOX3 antibody in Nthyi-ori-3 cell. **(D)** The list of top 20 proteins with the most significant differences by mass spectrometry identification and analysis. ***P < 0.001, data were pooled from three independent experiments. SDS-PAGE, SDS-polyacrylamide gel electrophoresis; RIP, RNA immunoprecipitation analysis; qPCR, quantitative polymerase chain reaction; RT-PCR, reverse transcription polymerase chain reaction.

To further explore the underlying molecular mechanism of SNHG9 regulation in PTC, we conducted an RNA pull-down assay using Nthy-ori-3 cells, in which silver staining showed significantly stronger bands at 110 kD, 60-40 kD, 34 kD and 20 kD compared with the control (**Figure 4A**). We further analyzed these bands by mass spectrometry (**Figure 4D**), and YBOX3 was among the top candidates obtained. YBOX3 can bind to mRNAs containing the sequence 5’-UCCAUCA-3’, stabilize the structure of the mRNA, and regulate the translation of the protein. The interaction between SNHG9 and YBOX3 identified by RNA pull-down mass spectrometry was further validated by Western blotting using an anti-YBOX3 antibody (**Figure 4B**), and we confirmed this interaction by RIP-qPCR assays. When compared to normal IgG, YBOX3 resulted in a significant enrichment of SNHG9 in Nthy-ori-3 cells (**Figure 4C**). RT-PCR result showed the length of SNHG9 determined by RIP experiment was about 470bp.

### SNHG9 Interferes With YBOX3 and Inhibits P21 Expression Through PTC Exosomes and Regulates Nthy-ori-3 Cell Autophagy and Apoptosis

A previous study found that the YBOX3 protein can bind to the 5’-UCCAUCA-3’ motif of the P21 mRNA under stress conditions, stabilize the mRNA and promote P21 protein expression (15), thereby preventing cells from apoptosis and maintaining cell survival. Therefore, we speculated that SNHG9 may regulate YBOX3 expression and further affect P21 to modulate apoptosis and autophagy.

**Figure 5:**
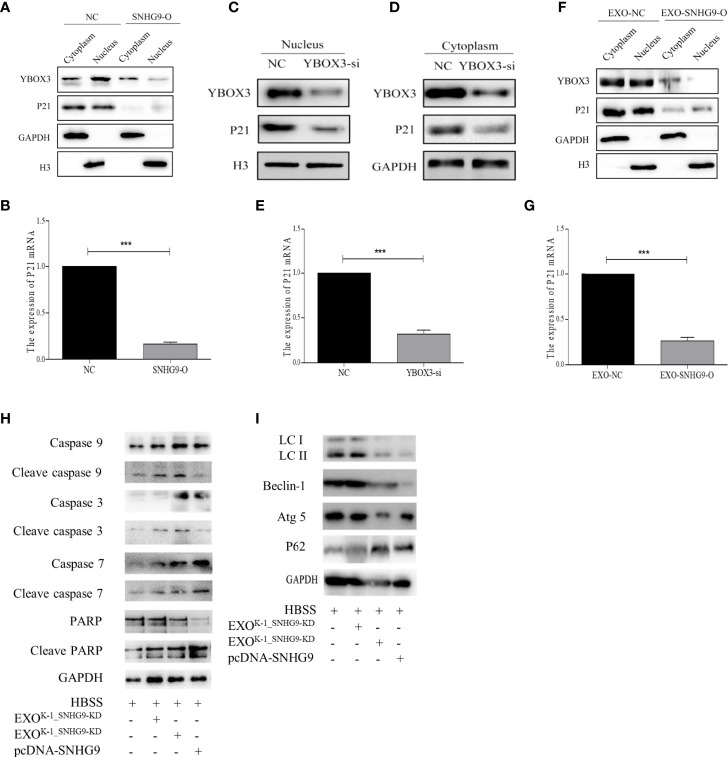
SNHG9 interferes with YBOX3 and inhibits P21 expression through PTC exosomes and regulates Nthy-ori-3 cell autophagy and apoptosis. **(A)** SNHG9 overexpression could inhibit YBOX3 and P21 protein expression level in both the cytoplasm and nucleus tested by Western blotting. The intracellular reference was GAPDH. The internal nuclear reference was H3. **(B)** P21 mRNA expression was decreased by SNHG9 overexpression in Nthy-ori-3 cell tested by qPCR. **(C, D)** Both P21 and YBOX3 protein expression were downregulated in cytoplasm and nucleus of Nthy-ori-3 cell after transfection of small interfering RNA for YBOX3. **(E)** P21 mRNA expression was decreased in Nthy-ori-3 cell after transfection of small interfering RNA for YBOX3. **(F)** Both P21 and YBOX3 protein expression were downregulated in cytoplasm and nucleus of Nthy-ori-3 treated with exosomes of K-1_SNHG9-O. **(G)** P21 mRNA expression was decreased in Nthy-ori-3 cell treated with exosomes of K-1_SNHG9-O. **(H)** Apoptosis pathway related protein expression in Nthy-ori-3 cells overexpressing SNHG9 or treated with K-1_SNHG9-O. **(I)** Autophagy pathway related protein expression in Nthy-ori-3 cells overexpressing SNHG9 or treated with K-1_SNHG9-O. ***P < 0.001, data were pooled from three independent experiments. PTC, papillary thyroid cancer; O, overexpression; qPCR, quantitative polymerase chain reaction.

We first used transient transfection to overexpress SNHG9 in Nthy-ori-3 cells and then extracted cytoplasmic and nuclease proteins. After overexpressing SNHG9, significant reductions in YBOX3 protein levels in both the cytoplasm and nucleus were observed (**Figure 5A**). Furthermore, both P21 mRNA and protein levels were decreased in the SNHG9 overexpression group (**Figures 5A, B**). The results indicate that SNHG9 negatively regulates YBOX3 and P21.

To further confirm that P21 downregulation is related to YBOX3, we constructed a small interfering RNA for YBOX3 to knock down its expression. Western blotting and qPCR results showed that P21 protein and mRNA expression levels were significantly decreased after interfering with YBOX3 expression (**Figures 5C–E**).

We extracted exosomes of K-1_SNHG9-O and cocultured them with Nthy-ori-3 cells. Compared with the control group, exosomes overexpressing SNHG9 resulted in downregulation of YBOX3 and P21 protein levels in the cytoplasm and nucleus (**Figure 5F**). P21 mRNA levels were also decreased in Nthy-ori-3 cells treated with exosomes overexpressing SNHG9 (**Figure 5G**). These data indicate that SNHG9 interferes with YBOX3 protein through exosomes and inhibits P21 expression.

We then detected apoptosis and autophagy-related protein expression after overexpressing SNHG9 and treating Nthy-ori-3 cells with PTC exosomes overexpressing SNHG9. As presented in **Figure 5H**, levels of the caspase apoptosis pathway proteins caspase 3, caspase 9, caspase 7, and PARP and their respective cleaved forms were increased after treatment with SNHG9-overexpressing plasmids or exosomes, suggesting that the cells entered apoptosis. In addition, expression of the autophagy pathway proteins Beclin-1 and Atg5 was downregulated, the P62 protein was aggregated, and LCII/LCI protein levels were decreased (**Figure 5I**), indicating that Nthy-ori-3 cell autophagy was inhibited.

## Discussion

SNHG9 (small nucleolar RNA host gene 9) is a lncRNA with a coding gene located on chromosome 17 and a total length of approximately 416 bp. There are few basic research reports on SNHG9 to date. In this study, we carried out a preliminary exploration of lncRNAs related to PTC cells and their exosomes. We used lncRNA chip technology for high-throughput screening, and we obtained the differential expression profiles of lncRNAs in the exosomes of TPC-1, K-1 and Nthy-ori-3 cell lines by bioinformatics analysis; SNHG9 was at the top of the list, indicating its potential role in PTC tumorigenesis. GO analysis results show that SNHG9 participates in DNA damage repair, apoptosis signal pathway regulation, cell autophagy and other cell processes. More importantly, we found that the distribution of SNHG9 exhibited significant exosomal enrichment. This result suggests that as a carrier of key molecules for cell to cell information regulation, exosomes are involved in the pathogenesis of PTC and that SNHG9 may be one of the key molecules acting through unique molecular mechanisms, participating in the pathogenesis and progression of PTC.

Autophagy is a cell-selective process accompanied by different protein degradation rates and sites (16, 17). Autophagy is tightly regulated under normal conditions, helping cells to maintain balance in synthesis and degradation of cellular components (18). However, in the context of tumors, autophagy can help cells cope with a variety of exogenous stimuli, such as nutrient deficiency, cell density load, and hypoxic oxidative stress. Overall, it is an important mechanism for cells to maintain homeostasis (19, 20).

Apoptosis is a type of programmed cell death during which DNA fragmentation occurs, the cell begins to shrink (16) and become rounded, nuclear chromatin condenses, the nucleus shrinks, a vacuolated cell membrane appears, and apoptotic bodies (including some organelles, ribosomes, and nuclear fragments) form. The relationship between autophagy and apoptosis is complex (21–23). Both processes play critical roles in controlling cell death and survival (24). They can be stimulated by the same stresses (24). In response to stress or damage, autophagy can keep cells viable and metabolically inert, allowing cellular repair and escape from further damage. The occurrence of autophagy can inhibit apoptosis, thereby protecting cells from stress or a lack of energy metabolism due to premature death (25). Inspired by this, after overexpressing SNHG9 in Nthy-ori-3 cells, we examined apoptosis by flow cytometry and found that the number of cells overexpressing SNHG9 undergoing apoptosis was significantly increased compared with the control group. Furthermore, we extracted the respective exosomes from the autophagy group described above, treated Nthy-ori-3 cells with these exosomes and detected apoptosis by flow cytometry. According to the results, the level of apoptosis in the autophagy inhibition group increased to varying degrees. In a coculture system, we used TUNEL staining to detect apoptosis and found significantly stronger staining in the autophagy inhibition group than in the control group. The above results indicate that PTC cells can transmit SNHG9 to surrounding normal cells through exosomes, thereby inhibiting autophagy in normal cells, interfering with their ability to adapt to stress, hyponutrition or hypoxia, and disrupting the internal environment and promoting apoptosis.

We applied RNA pulldown and mass spectrometry to identify target proteins of SNHG9. GO and KEGG analyses and query of the 20 most distinct proteins in a database revealed the transcription factor YBOX3. RIP assays confirmed that SNHG9 is indeed able to bind to the YBOX3 protein, and Western blotting confirmed that SNHG9 negatively regulates YBOX3 in Nthy-ori-3 cells.

It has been reported that YBOX3 is activated in cells under stress and that it binds to the 5’-UCCAUCA-3’ end of the P21 mRNA to stabilize its structure, promote its translation, and increase P21 protein expression. P21 is an important member of the cyclin-dependent kinase inhibitor family, and in the presence of DNA damage, P21 arrests the cell growth cycle, thereby protecting cells from apoptosis (26). It has been reported that P21 can promote degradation of activated caspase 3 and inhibit activation of the apoptotic pathway, preventing apoptosis and promoting survival (27). However, some researchers have emphasized that in the case of ROS induction, translation of the P21 protein is enhanced and activates the p38 MAPKα pathway, which increases expression of Beclin-1, promotes autophagy and maintains the stability of the intracellular environment (28). Therefore, we confirmed that SNHG9 interferes with YBOX3 protein expression through PTC exosomes, negatively regulates P21, inhibits autophagy, and promotes apoptosis under stress conditions.

Our study suggests that SNHG9 acts as a PTC exosome-enriched lncRNA and regulates Nthy-ori-3 cell autophagy and apoptosis by interfering with the YBOX3 and P21 pathways. Nevertheless, the observations that SNHG9 targets YBOX3 to regulate autophagy and apoptosis in normal cells were obtained *in vitro*, and further verification in animal models is needed. More follow-up studies are necessary to further clarify how SNHG9 induces YBOX3 degradation.

## Data Availability Statement

The datasets presented in this study can be found in online repositories. The names of the repository/repositories and accession number(s) can be found below: https://www.ncbi.nlm.nih.gov/, SUB8325912.

## Author Contributions

DW and W-lL performed cell line culture, RNA-pull down, RIP assay, statistical analysis, and wrote the paper. Z-wL performed FUSCC human tissues collection and helped write the paper. Y-mC performed qPCR and Western blotting. Q-hJ provided human PTC tissues and technical support. W-jW designed this project and supervised this project. All authors contributed to the article and approved the submitted version.

## Funding

This work was supported by the National Natural Science Foundation of China (81772854, 81572622 to Q-hJ, 82002381 to W-lL) and Natural Science Foundation of Shanghai (19ZR1410900 to W-jW).

## Conflict of Interest

The authors declare that the research was conducted in the absence of any commercial or financial relationships that could be construed as a potential conflict of interest.

## Supplementary Material

Supplementary Figure 1 Validation of cell exosomes extraction. **(A)** Cell exosome marker CD9 and CD63 showed successful cell exosome extraction by Western blotting. **(B)** The shape of exosomes was observed by transmission electron microscopy.

Supplementary Figure 2 **(A, C)** Overexpression efficiency of SNHG9 in TPC-1, K-1 and Nthy-ori-3 cell lines confirmed by qPCR. **(D, F)** Knockdown efficiency of SNHG9 in TPC-1 and K-1 cell lines confirmed by qPCR.

Supplementary Figure 3 SNHG9 could inhibit Nthy-ori-3 cell autophagy through TPC-1 exosomes. **(A, C)** TPC-1^SNHG9-O/^TPC -1^SNHG9-KD^ exosomes could inhibit/promote Nthy-ori-3 cells autophagy detected by mRFP/mCherry-GFP-LC3B autophagy flow. Yellow spots indicate autophagosomes and red spots indicate autophagic lysosomes. **(B)** P62 was significantly increased in TPC-1^SNHG9-O^ exosome treated Nthy-ori-3 and was downregulated in TPC-1^SNHG9-KD^ exosome treated Nthy-ori-3 cells by Western blotting. **(D)** The impact of TPC-1^SNHG9-O/^TPC-1^SNHG9-KD^ exosomes on Nthy-ori-3 cell autophagy was decreased after TPC-1 cell exosomes secretion was inhibited by GW4869. **(E)** P62 protein change after TPC-1 cell exosomes secretion was inhibited by GW4869.

Supplementary Figure 4 SNHG9 could promote Nthy-ori-3 cell apoptosis through TPC-1 exosomes. **(A, B)** TPC-1^SNHG9-O/^TPC-1^SNHG9-KD^ exosomes could promote/inhibit Nthy-ori-3 cells apoptosis detected by flow cytometry.
