# Supplementary figures and images for: SNHG9, a Papillary Thyroid Cancer Cell Exosome-Enriched lncRNA, Inhibits Cell Autophagy and Promotes Cell Apoptosis of Normal Thyroid Epithelial Cell Nthy-ori-3 Through YBOX3/P21 Pathway
Source: Front Oncol. 2021 May 4;11:647034. doi: 10.3389/fonc.2021.647034 (PMC8129558; doi:10.3389/fonc.2021.647034)

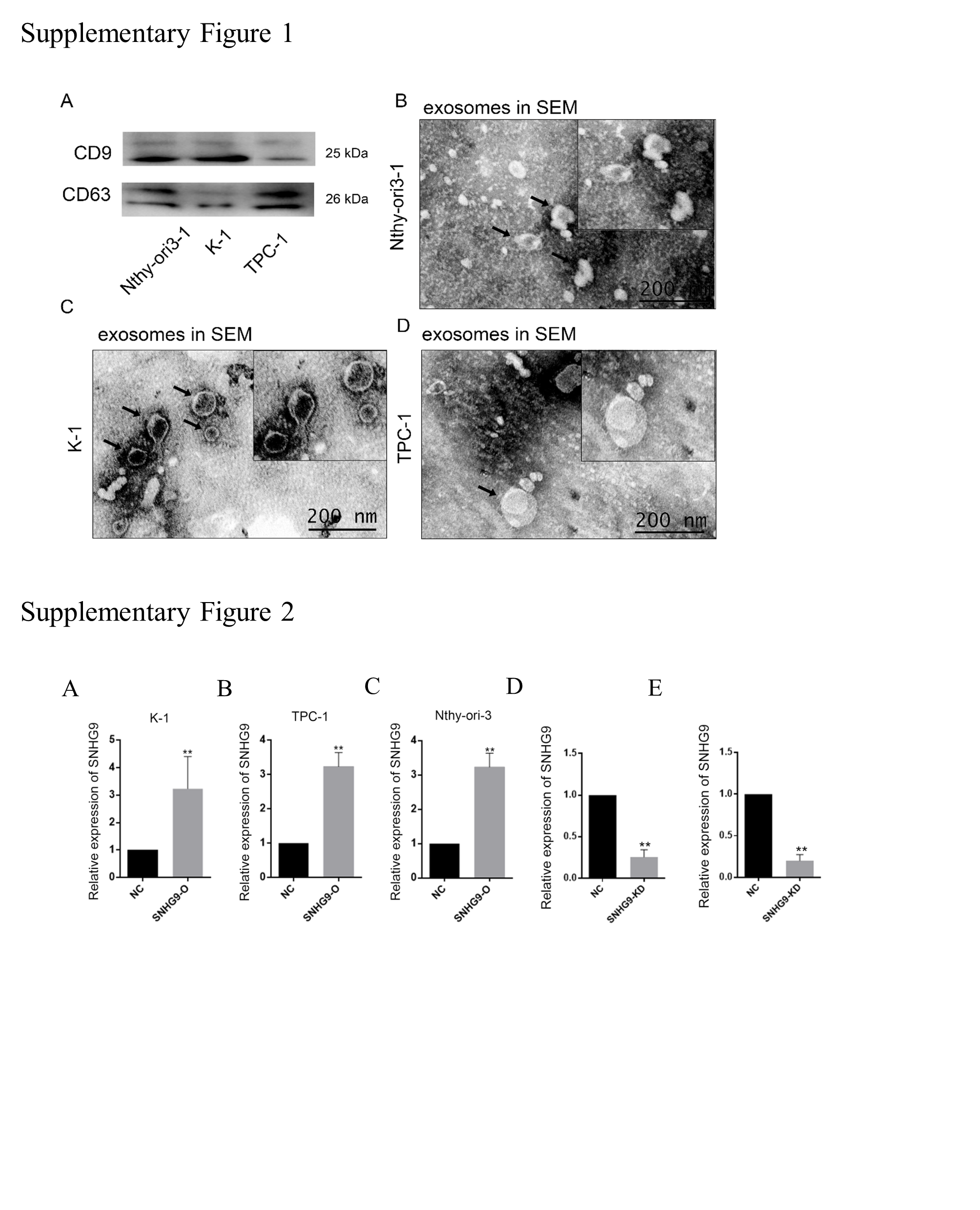

Supplement: Supplementary Figure 1 — Validation of cell exosomes extraction. (A) Cell exosome marker CD9 and CD63 showed successful cell exosome extraction by Western blotting. (B) The shape of exosomes was observed by transmission electron microscopy. [file Image_1.tif]

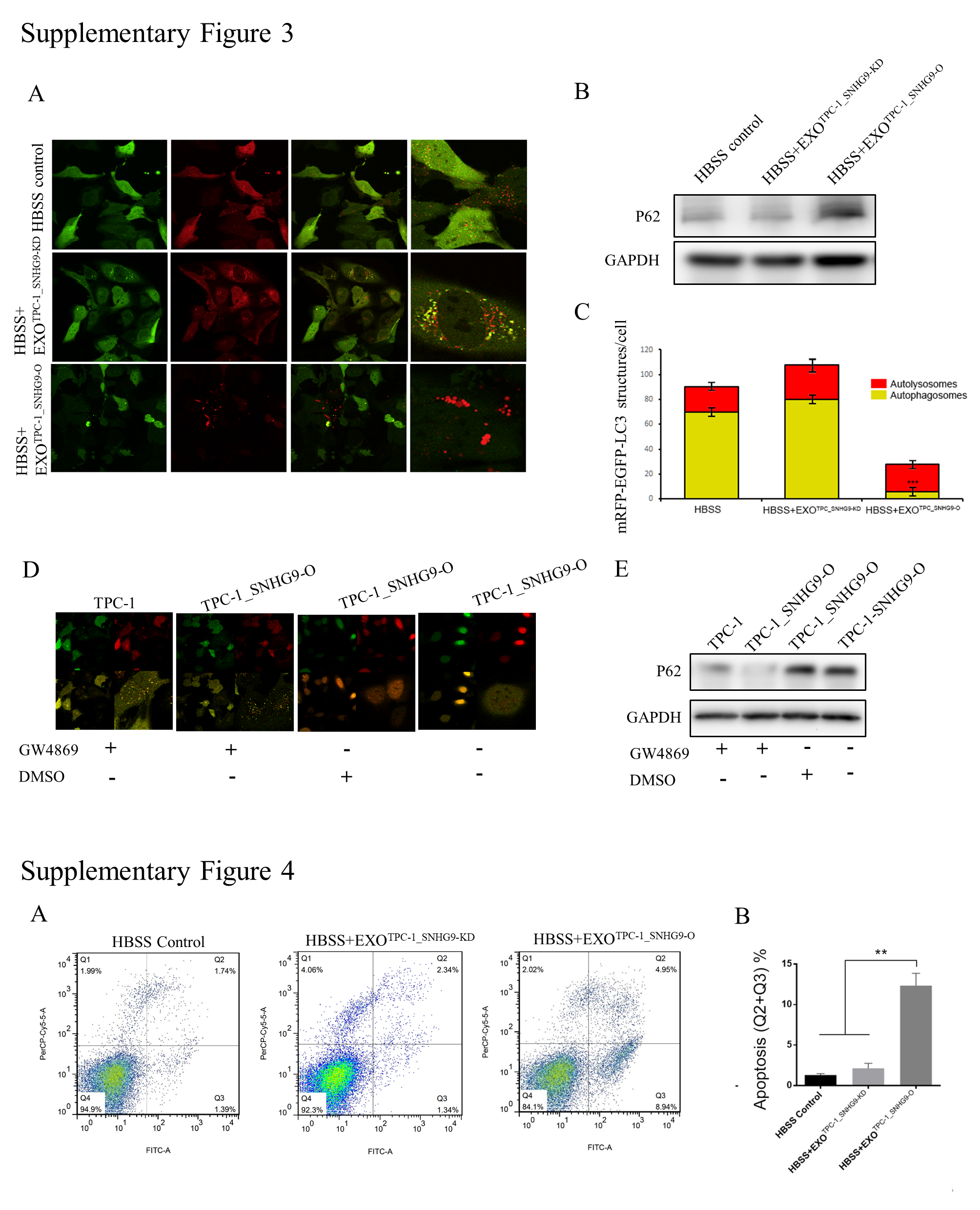

Supplement: Supplementary Figure 3 — SNHG9 could inhibit Nthy-ori-3 cell autophagy through TPC-1 exosomes. (A, C) TPC-1SNHG9-O/TPC -1SNHG9-KD exosomes could inhibit/promote Nthy-ori-3 cells autophagy detected by mRFP/mCherry-GFP-LC3B autophagy flow. Yellow spots indicate autophagosomes and red spots indicate autophagic lysosomes. (B) P62 was significantly increased in TPC-1SNHG9-O exosome treated Nthy-ori-3 and was downregulated in TPC-1SNHG9-KD exosome treated Nthy-ori-3 cells by Western blotting. (D) The impact of TPC-1SNHG9-O/TPC-1SNHG9-KD exosomes on Nthy-ori-3 cell autophagy was decreased after TPC-1 cell exosomes secretion was inhibited by GW4869. (E) P62 protein change after TPC-1 cell exosomes secretion was inhibited by GW4869. [file Image_2.tif]
